# A “Curriculum of Information Needs” of Parents of Children With Chronic Constipation

**DOI:** 10.1177/00099228251395563

**Published:** 2025-12-01

**Authors:** Sadia Zaman, Tabitha Ashley-Norman, Jonathan Sutcliffe, Peter Cartledge

**Affiliations:** 1 School of Medicine, University of Leeds, Leeds, UK; 2 Leeds Teaching Hospitals NHS Trust (LTHT), Leeds, UK; 3 Leeds Community Healthcare NHS Trust, Leeds, UK; 4 Kent and Medway Medical School (KMMS), Canterbury, UK

**Keywords:** constipation, patient education as topic, child, pediatrics, qualitative research

## Abstract

Chronic idiopathic constipation carries a significant burden on children and their families. Despite existing treatment guidelines, adherence and outcomes remain poor, with gaps in parental understanding contributing to these outcomes. This study aimed to develop a “curriculum of information needs” for parents of children with chronic constipation. This is a qualitative study which employed 15 semi-structured interviews with parents and health care professionals, in the United Kingdom. Thematic analysis of data produced 4 themes, with the primary study objective described in the first theme of a “curriculum of information needs.” This curriculum encompasses key topics, such as; understanding constipation, behavioral strategies for management, and medication use, providing a framework to enhance parental knowledge and understanding of chronic constipation. Future efforts could integrate findings into designing accessible resources, including digital platforms and Artificial Intelligence-driven treatment tools, to improve adherence and outcomes.

## Introduction

Children with chronic idiopathic constipation (CIC) carry a significant burden of disease, affecting overall quality of life. Professionals, and carers, supporting children with CIC recognize that this affects physical, behavioral, emotional, psychosocial, financial, schooling and social sequelae, along with financial burdens for children, families, and the health care system.^1-4^ Over one-third of patients with functional constipation develop chronic symptoms, often prompting secondary care referral attendance.^5^

Although prevention and treatment recommendations for CIC already exist, the evidence base remains limited.^6-8^ Poor satisfaction with treatments along with poor adherence in adults is mirrored in the pediatric population, with adherence of children treated markedly low at 37%.^9,10^ If treatments do improve outcomes in the real world, then outcomes will be affected by adherence. Furthermore, psychoeducation, improved engagement and communication will improve the feedback loop for clinicians and the confidence of families.

Psychoeducation teaches the knowledge and skills required for parents to understand and manage constipation.^11^ It is a blend of interventions, such as education and skills-based training, to help promote a collaborative approach between patients, their families and health care providers.^12^ Psychoeducation interventions include verbal information, written materials, web-sources, Apps or within forums.^13-17^ At the heart of this is a shift from paternalistic models of health care, placing patients as passive recipients of care, to more collaborative, patient-centered, communicative approaches.^7,14^ Thompson’s systematic review described the “precarious footing” experienced by families, when dealing with limited knowledge and managing their child’s constipation and confirms the need to improve insight into parents’ preferences for education resources and how to access them.^17-19^ Therefore, this study aimed to produce a “*curriculum of information needs”* to enable caregivers to better care for their children with constipation. We wanted to explore the views of parents and caregivers of children with constipation presenting to professionals in the United Kingdom, who needed information to better understand and manage their child’s idiopathic constipation. Any caregiver (including grandparents, foster and adoptive parents) was included, along with the child, where age applicable, as a family unit described as “parents.” The insights from this study could be used for the design of future resources to help improve adherence and outcomes for treatment of CIC.

## Methods

### Study Design

Reporting for this study adheres to the COREQ checklist for qualitative studies *(see supplementary file- COREQ checklist)*.^20^ Qualitative methods employing 1-to-1 semi-structured interviews (SSIs), between January and July 2022, facilitated collection of accounts from participants. Other methods, such as surveys, were not employed, as they would have limited the response range and subsequently would have restricted exploration of findings.

### Study Site

The primary researchers were based at a Community Healthcare NHS trust, University, and UK tertiary pediatric surgical center.

### Study Participants

The study included a “panel of experts” in the field of childhood constipation, separated into 2 main groups; those with “lived experience” (parents of children with constipation, diagnosed over 1 year ago) (group 1), or health care professionals (HCPs) with expertise in childhood constipation (group 2). All participants were over the age of 18 and had access to a computer with video conferencing software. Since this work was completed without funding, we were unable to provide support to include participants with significant learning difficulties or communication needs, including the need for an interpreter. Therefore, these groups were excluded from the study.

### Researcher Gender, Credentials, Occupation, Experience, and Training

Interviews were conducted by either 1 of 2 female fourth-year medical students (TAN & SZ), with no previous contact, knowledge or relationship with participants, apart from email contact to arrange the interview. Both interviewers had previous experience in conducting qualitative research. PC supervised and co-authored the work and is a male, consultant pediatrician, trained in pediatrics in the United Kingdom. One supervisor, PC was the clinical lead for constipation at Leeds Community NHS Trust and the other, JS is a former member of the relevant NICE guideline development group and a Pediatric Colorectal Surgeon. Both supervisors had qualitative research experience.

### Recruitment, Sampling and Enrolment

#### Purposive sampling

Non-probability purposive sampling was conducted to include a wide range of stakeholders. For group 1, researchers aimed to recruit at least 1 parent from the following categories: male, female, parent of a child with special educational needs (SEN), parent from a “difficult to access” group (social deprivation, low health literacy). For group 2 participants (HCPs), researchers aimed to recruit at least 1 continence nurse, pediatrician and pediatric surgeon.

#### Saturation

Saturation was predefined as the point at which there was no further emergence of new codes.^21^

#### Recruitment

Participants were recruited from across the United Kingdom. To recruit parents (group 1), the study was advertised via the HealthUnlocked forum (https://healthunlocked.com/eric) in collaboration with a national charity supporting children and the families of children with incontinence (ERIC). Researchers also contacted eligible HCPs (group 2), through established professional networks and subsequent snowball sampling.

#### Enrolment

Enrolment was undertaken in line with University of Leeds (UoL) standards on approaching and recruiting research participants. Participants who expressed an interest in the study were sent a participant information sheet and consent form, digitally via email *(see supplementary files—information sheets and consent forms)*. The study was advertised on HealthUnlocked; therefore, the number of views of the invite was not monitored. Nonparticipation rates of those who expressed an interest were recorded.

### Study Procedures

#### Interviews

An interview guide was constructed for each group and used to conduct interviews *(see supplementary files—interview guides).* Interviews were undertaken remotely via Zoom video conferencing. At the start of each interview, interviewers verbally communicated a summary of the research objectives and aims and confirmed verbal consent with the interviewee. Interviewers used a UoL Zoom account, conducting the interviews (SSIs) in a private room, with no third-parties present. Participants were also encouraged to be in a private room in the interest of maintaining confidentiality. No field-notes were taken during interviews. Repeat interviews were not offered to participants.

#### Recording and transcription

Video and audio recordings of interviews were taken, and Zoom software was used to automatically transcribe the interviews. Transcription was automatically undertaken immediately after each interview, utilizing Zoom software. Transcripts were checked by the interviewer for accuracy. Transcripts were not given to participants for review.

#### Confidentiality

Participants were pseudo-anonymized through allocation of a Unique Patient Identifier (UPI).

#### Pilot interview

One pilot interview was carried out to assess the quality and comprehensiveness of the interview guide, interview timing, the consent process, transcription, and recording. None of the data from this interview was analyzed or used.

### Analysis

#### Approach

A pragmatic approach was taken to qualitative coding and thematic analysis, as described by Kiger and Varpio.^22^ Familiarization of data (step 1) was carried out during the checking of transcriptions.

#### Software

The transcripts were uploaded onto Microsoft-Excel, to digitally support coding and analysis, with each line of dialogue representing one data-point.^23^ Initial code generation (Step 2) was carried out through the assignment of codes to each data-point. An audit chain was maintained for transcription, coding, and analysis.

#### Coding

Each transcript was analyzed independently by 3 researchers (TAN, SZ and PC) resulting in the whole dataset being analyzed 3 times. Coding was inductive, with de novo codes emerging from the data. Researchers ensured that the codes encompassed discrete and nonoverlapping features of the data and to facilitate the production of the curriculum of information needs for parents. Coding was undertaken in blocks of 2 to 3 interviews, separated into group 1 and group 2. SZ initially coded group 1, TAN coded group 2. Subsequently, SZ re-coded for group 2 and TAN re-coded for group 1. Subsequently, comparison of the code assignment was carried out to resolve any discrepancies in analysis. A third researcher (PC) cross-checked the coding and acted as an arbiter on nonagreement. Participants were not offered the opportunity to provide feedback on coding or analysis.

#### Thematic analysis

Analysis was undertaken with the complete body of transcripts. Themes were derived through comparing associations between the codes (Step 3 of Kiger) and grouping them into broader themes along with frequencies of codes within the theme.^22^ Themes were reviewed and named to ensure that codes were appropriately distributed amongst their respective themes, resulting in a rich and descriptive thematic framework (Steps 4 & 5). Finally, the thematic framework was used in the manuscript write-up (Step 6).

#### Ethics

Ethical approval was sought for this study, which aimed to produce generalizable findings. Approval was approved by the University of Leeds ethics panel, (MREC 21-018). Our study did not require NHS Research Ethics Committee approval.

#### Consent

Participants were emailed a participant information sheet and consent sheet over 48 hours before the interview, and were informed that participation is voluntary. Verbal consent was gained in accordance with UoL protocol. Verbal consent was deemed as the most appropriate method, as written consent would have required participants to print and send the completed consent form, which could disadvantage those with reduced digital literacy.

#### Conflict of interest

Authors report no conflicts of interest.

#### Data governance

Data storage was undertaken in keeping with UoL principles regarding Collecting Research Data. Zoom recordings were stored on UoL OneDrive, and were deleted following completion of interview transcriptions. Transcripts and consent forms were stored for 3 years on a secure UoL OneDrive, only accessible by members of the research team. Coding of the transcripts was inputted onto a password-protected Excel spreadsheet and stored on the UoL OneDrive. Communication between the researchers was facilitated using secure email (@nhs.net or @leeds.ac.uk).

## Results

### Participants and Interviews

**Figure 1.**
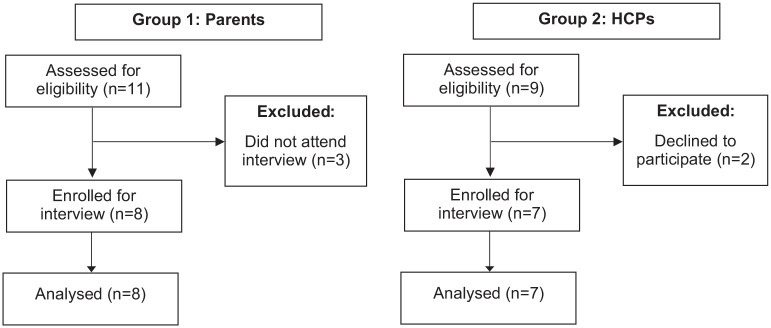
Flow diagram of participant enrollment.

Fifteen interviews were undertaken. Mean length of interviews was 23 minutes (shortest 12 mins; longest 43 mins). Researchers recruited 7 parents for group 1 and 8 professionals for group 2, at which point data saturation was reached (Figure 1). Parents from all purposive sampling categories were recruited, except for one from a “difficult to access” group. Seven parents were mothers, 1 was a father, and 1 parent had a child with suspected autism and special educational needs. The mean age of the participant’s children was 5.3 years (range = 4-10 years), diagnosed with constipation for a mean of 3.0 years (range = 1-10 years). Group 2 HCPs were recruited from each purposive category: continence nurses (n = 3), general pediatrician (n = 2), community pediatrician (n = 1) and pediatric surgeon (n = 1).

### Codes and Thematic Framework

A total of 71 codes developed inductively during the analysis, falling within 4 themes, derived from the data, namely, (1) curriculum of information needs; (2) sources of current information; (3) format / media of information; and (4) barriers to education. As the primary objective of this study was to describe a “curriculum” of information needs, only Theme 1 is presented here, with Themes 2 to 4 results being presented, for article brevity, as supplemental data.

### Our “Curriculum of Information Needs”

Our participants described 30 topics that they deemed important for parents. Within the curriculum of information needs there were 5 subthemes (Table 1), namely,

*Understanding constipation (13 topics)*

I literally got the box, the label the dose and no further information, no patient information about constipation itself and so, in terms of what I would expect or would want as a parent would be. Perhaps they suggested dietary things like increasing fruits, vegetables and fluids, etc, but none of that was ever given to me in writing. (Parent)

*Management—investigations (single topic)*

When we talk about causes [we should discuss that] it’s very unlikely that we have to investigate further, but we do occasionally do blood tests and X-rays . . . if we’re not entirely sure it is basic constipation. (HCP)

*Management—behavioral strategies (4 topics)*

I don’t think we’ve been taken seriously at GP level and the nursery, in terms of toilet training, [it] was always “oh she’ll get over it, she’ll get over it,” but she’s now six in school and, well pooing is a big issue still, she will not poo in school. (Parent)

*Management—support (2 topics)*

How can we [manage] this at home with your GP and when do we say “this isn’t working” and we move onto the next step. (Parent)

*Management—medication (10 topics)*

There’s something else that was not really available at the minute is, some sort of video about the use of rectal therapies and obviously it’s not available for very good reasons because you know videos about bottoms [are] a bit taboo, but it would be really useful to have some sort of video about how to give rectal therapies. (HCP)

**Table 1.** Theme 1. Coding, to Provide Our “Curriculum of Information Needs.”

	Subtheme: 1.1 Understanding constipation	Frequency of code
1.1.1	Fluids and diet	48
1.1.2	Definition of constipation	43
1.1.3	Signs and symptoms	36
1.1.4	Disease timescale	23
1.1.5	Causes of constipation	23
1.1.6	Soiling / overflow	22
1.1.7	Potty / toilet training	14
1.1.8	Basic anatomy of the body	13
1.1.9	Prevention	9
1.1.10	Information for schools	7
1.1.11	Other toileting problems (e.g. wetting) related to constipation	7
1.1.12	Types of stool / Bristol Stool Chart	6
1.1.13	Stool withholding	4
	Subtheme: 1.2 Management—investigations	
1.2.1	Investigations	5
	Subtheme: 1.3 Management—behavioral strategies	
1.3.1	Toileting habits	39
1.3.2	How to cope at school	12
1.3.3	Behavioral treatment strategies	4
1.3.4	Exercise	2
	Subtheme: 1.4 Management—support	
1.4.1	When and how to access help	17
1.4.2	General parenting support	17
	Subtheme: 1.5 Management—medication	
1.5.1	How medications work	61
1.5.2	Tips for how to get child to take medication	27
1.5.3	Medication—disimpaction	16
1.5.4	Compliance	12
1.5.5	Second line treatments	11
1.5.6	Medication—how to make/take them	9
1.5.7	Taking tablets	4
1.5.8	Titrating doses of medications	3
1.5.9	Medication—treatment timescale	3
1.5.10	Medication—side-effects	2

## Discussion

This study aimed to produce a “curriculum of information needs” that parents need to care for their children with constipation. Employing qualitative methods, we describe a curriculum of 5 subthemes, comprising 30 topics.

Improvements to health through psychoeducation has been shown to be effective in providing significant improvements in parent satisfaction, knowledge, and Quality of Life (QoL).^24,25^ Parents want information content that is comprehensive, consistent and evidence-based.^15^ Effective information can come in a range of sources, for example, verbal information, written materials, web-sources, Apps, or within forums.^13-17,26^

Parents have quite specific information needs and Thompson’s systematic review described the “precarious footing” experienced by families, when dealing with limited knowledge and managing their child’s constipation.^17^ These challenges pose risks to the stability of family relationships and the effectiveness of treatment plans. Uncertainty is a recurring theme in parents’ experiences, encompassing their grasp of the diagnosis, the execution of intricate interventions, their navigation of the health care system, and their management of family relationships. Parents supporting a child with constipation typically possess a mix of accurate information and misconceptions regarding the condition and its treatments. The presence of information gaps and misinformation has a detrimental impact on parental confidence and their ability to make decisions regarding their child’s health, and ultimately outcomes for their child.^3,17^ Consequently, parents frequently find themselves lacking a solid foundation for caregiving.^15,17^ Thompson goes on to describe the “(mis)understanding” of parents regarding the condition, and symptoms of constipation often persist for months or even years before families seek help. Furthermore, parents remember enduring frustratingly long periods before receiving a clear and explicitly communicated diagnosis. It is common for parents to recount numerous visits to health care providers in an attempt to better understand their child’s symptoms.^17^ These are frequent visits that deplete resources in stretched health care settings. Therefore, what are the information needs of parents? We have found 5 key subthemes, with a total of 30 topics (Table 1).

The Subtheme of “Understanding constipation” included 13 topics related to the diagnosis, ranging from anatomy, signs, symptoms, to stool withholding, potty training, and timescales (Table 1). Prior research has indicated that parents tasked with making decisions regarding their child’s health are often left uninformed about the underlying causes, and they frequently receive inaccurate information concerning dietary modifications and the expected course of constipation.^15,17^ Understanding is also required to initiate or adhere correctly to medications, for example, parents often misunderstand overflow fecal incontinence as an intentional behavior or as a sign of “over treatment.”

The subtheme of “Management—behavioral strategies” included essential information needs on what normal toileting habits are, and how to establish them through behavioral management strategies. Medications have been shown to be effective for treating constipation.^6,7^ However, behavioral strategies are at the heart of good management and long-term prevention and therefore families need a range of information resources to support this.^4,6,7,17^

Within the subtheme of “Management—medication” we found 10 parental information needs on medications, ranging from how the medications work, how to prepare them and give them, how to titrate the dosages to response, and any side-effects they can expect. Writing a prescription is relatively straightforward and there is clear guidance, for professionals, on first-line, effective strategies.^6,7^ However, for parents with little or no experience, administering, titrating and complying with unpleasant medications is challenging. To exacerbate this, parents are frequently concerned about medication use.^17^

It’s important to consider who should produce and provide education: Our participants reported wanting information from General Practitioners (GPs), continence nurses, pharmacists, schools, the charity sector, forums and support groups, along with other online resources and signposting from professionals, which echoes findings of previous literature.^13-17^

In terms of the implications and future work related to our findings: By filling the information gap of families, parental and child experience and outcomes will hopefully be improved. There is no “one size fits all” in terms of information needs of parents. Some children require a short course of treatment, with minimal parental education, with good outcomes. These families would generally not require a full “curriculum” of education, but having the full curriculum of education resources available would be beneficial. Other children have chronic symptoms, nonresponsive to treatment, or poor compliance/tolerance, requiring more parental education. In this case the full curriculum of information resources is likely to be required by parents. Our study used qualitative methods; therefore, generalizability is an important consideration, with our results best suited to the UK setting and context. We would advocate that implementation in this setting should be with the key national stakeholder for parental support, which is currently the UK charity ERIC which is widely used by parents and professionals. Providing face-to-face information to parents, from a professional, is a valuable activity, however, it is time-consuming, costly, and not often well-timed. Mobile phone applications and Artificial Intelligence (AI) offer further opportunities to implement the curriculum described. Creating resources that assess parental understanding and “fill the gap” would be well received, and with the prevalence of constipation being high, investment would be worth making. AI could offer the hope of combining bespoke information giving, with timely titrating of medication.^27,28^ Assessing the validity of the items within the curriculum itself would likely require further qualitative work. Whereas measuring the impact of implementation of psychoeducation via tools such as AI or Apps would most benefit from quantitative assessments. For use in other settings and contexts, employing consensus methods, such as Delphi methods, using our curriculum as a first-stage, could proactively adapt a curriculum for the needs of a local population or service.^29,30^

Our study has several limitations. We recruited a wide range of experts, employed qualitative methods with relatively small sample sizes, which limited the generalizability of findings. Several secondary data points were not obtained, such as demographic information relating to the educational level and socio-economic status of families. Further information, such as the criteria and health care setting through which the diagnosis of constipation was given, was also not collected. These data points may have provided a richer insight into the diversity of our sample population. Our study may have incurred sampling bias, due to the method of recruitment being through ERIC’s HealthUnlocked forum, mainly used by parents struggling with constipation. The use of Zoom video conferencing may have excluded nondigital literate participants. Therefore, these parents are more likely to be those who advocate for their children and have more resources and available time to search for answers. Lastly, our sample did not include “underserved” participants (e.g. parents with learning disabilities) as this study did not have the funding to support these groups. During purposive sampling design we did not include a GP, which, in hindsight, was an important omission.

## Author Contributions

**Sadia Zaman:** Conceptualization, Data Curation, Investigation, Formal Analysis, Methodology, Writing—Original Draft

**Tabitha Ashley-Norman:** Conceptualization, Data Curation, Investigation, Formal Analysis, Methodology, Writing—Original Draft

**Jonathan Sutcliffe:** Conceptualization, Writing—Review and Editing, Supervision

**Peter Cartledge:** Conceptualization, Data Curation, Software, Methodology, Writing—Review and Editing, Formal Analysis, Project Administration, Supervision

## Supplemental Material

sj-docx-1-cpj-10.1177_00099228251395563 – Supplemental material for A “Curriculum of Information Needs” of Parents of Children With Chronic Constipation Supplemental material, sj-docx-1-cpj-10.1177_00099228251395563 for A “Curriculum of Information Needs” of Parents of Children With Chronic Constipation by Sadia Zaman, Tabitha Ashley-Norman, Jonathan Sutcliffe and Peter Cartledge in Clinical Pediatrics

sj-docx-5-cpj-10.1177_00099228251395563 – Supplemental material for A “Curriculum of Information Needs” of Parents of Children With Chronic Constipation Supplemental material, sj-docx-5-cpj-10.1177_00099228251395563 for A “Curriculum of Information Needs” of Parents of Children With Chronic Constipation by Sadia Zaman, Tabitha Ashley-Norman, Jonathan Sutcliffe and Peter Cartledge in Clinical Pediatrics

sj-docx-6-cpj-10.1177_00099228251395563 – Supplemental material for A “Curriculum of Information Needs” of Parents of Children With Chronic Constipation Supplemental material, sj-docx-6-cpj-10.1177_00099228251395563 for A “Curriculum of Information Needs” of Parents of Children With Chronic Constipation by Sadia Zaman, Tabitha Ashley-Norman, Jonathan Sutcliffe and Peter Cartledge in Clinical Pediatrics

sj-docx-7-cpj-10.1177_00099228251395563 – Supplemental material for A “Curriculum of Information Needs” of Parents of Children With Chronic Constipation Supplemental material, sj-docx-7-cpj-10.1177_00099228251395563 for A “Curriculum of Information Needs” of Parents of Children With Chronic Constipation by Sadia Zaman, Tabitha Ashley-Norman, Jonathan Sutcliffe and Peter Cartledge in Clinical Pediatrics

sj-pdf-2-cpj-10.1177_00099228251395563 – Supplemental material for A “Curriculum of Information Needs” of Parents of Children With Chronic Constipation Supplemental material, sj-pdf-2-cpj-10.1177_00099228251395563 for A “Curriculum of Information Needs” of Parents of Children With Chronic Constipation by Sadia Zaman, Tabitha Ashley-Norman, Jonathan Sutcliffe and Peter Cartledge in Clinical Pediatrics

sj-pdf-3-cpj-10.1177_00099228251395563 – Supplemental material for A “Curriculum of Information Needs” of Parents of Children With Chronic Constipation Supplemental material, sj-pdf-3-cpj-10.1177_00099228251395563 for A “Curriculum of Information Needs” of Parents of Children With Chronic Constipation by Sadia Zaman, Tabitha Ashley-Norman, Jonathan Sutcliffe and Peter Cartledge in Clinical Pediatrics

sj-pdf-4-cpj-10.1177_00099228251395563 – Supplemental material for A “Curriculum of Information Needs” of Parents of Children With Chronic Constipation Supplemental material, sj-pdf-4-cpj-10.1177_00099228251395563 for A “Curriculum of Information Needs” of Parents of Children With Chronic Constipation by Sadia Zaman, Tabitha Ashley-Norman, Jonathan Sutcliffe and Peter Cartledge in Clinical Pediatrics
