# Supplementary material for: A “Curriculum of Information Needs” of Parents of Children With Chronic Constipation
Source: Clin Pediatr (Phila). 2025 Dec 1;65(3):403–10. doi: 10.1177/00099228251395563 (PMC12864524; doi:10.1177/00099228251395563)
Supplement: sj-docx-1-cpj-10.1177_00099228251395563 – Supplemental material for A “Curriculum of Information Needs” of Parents of Children With Chronic Constipation [file sj-docx-1-cpj-10.1177_00099228251395563.docx]

Domain 1:

Item 1-8: See Methods, “Researcher gender, credentials, occupation, experience and training”

Domain 2:

Item 9: See Methods, “Approach”

Item 10-11: See Methods, “Recruitment”

and “Enrolment”

Item 12: See Results “Participants and

Interviews”

Item 13: See Figure 1

Setting

Item 14: See Methods “Student Site”

Item 15: See Methods “Interviews”

Item 16: See Results “Participants and interviews”

Data collection

Item 17-20: See Methods “Interviews”

Item 21: See results “Participants and interviews”

Item 22: See Methods “Saturation”

Item 23: See Methods “Recording and

transcription”

Domain 3

Data analysis

Item 24: See Methods “Coding”

Item 25: See Table 1

Item 26: See Methods “Thematic analysis”

Item 27: See Methods “Software”

Item 28: See Methods “Coding”

Reporting

Item 29-30: See Table 1

Item 31-32: See Table 1, Methods “Thematic analysis” and the Results section
