# Supplementary material for: A “Curriculum of Information Needs” of Parents of Children With Chronic Constipation
Source: Clin Pediatr (Phila). 2025 Dec 1;65(3):403–10. doi: 10.1177/00099228251395563 (PMC12864524; doi:10.1177/00099228251395563)
Supplement: sj-docx-5-cpj-10.1177_00099228251395563 – Supplemental material for A “Curriculum of Information Needs” of Parents of Children With Chronic Constipation [file sj-docx-5-cpj-10.1177_00099228251395563.docx]

Round-1 Interview Guide 1, for Caregivers in Group 1

**Study title:** The information needs of caregivers of children with chronic constipation: a Delphi study

**Interview Documentation** (to be filled in by the interviewer during the interview)

*Interview information:*

Interview number:

Participant code number:

Date of interview:

Duration of interview:

Interviewer name:

*Demographics:*

Caregiver role:

Age of child:

Special educational needs? : YES/NO

Length of time since diagnosis:

**Guide**

*Introduction:*

1. Interviewer to introduce self, and ask participant what they would like to be called for the purposes of the interview, reminding them that their name will be removed from the transcript.
2. Interviewer to remind participant of the study title: *The information needs of caregivers of children with chronic constipation: a Delphi study.*
3. Interviewer to give participant an opportunity to ask any questions about their participation in the study.
4. Interviewer to remind participant that they are not obliged to answer any questions and are free to stop the interview at any point.
5. Interviewer to explain that the recording of the interview will begin,.
6. Interviewer to press record.
7. Consent questions

*Section 1: Demographics:*

1. What is their role as a caregiver? (parent, guardian, sibling)
2. How old is their child?
3. Does their child have any special educational needs?
4. How long has their child been diagnosed with constipation, soiling or encoporesis?

*Section 2: Content of information which is currently provided*

We want to ensure that there is information on all the aspects of constipation that parents want to know about.

1. What do you think are the key topics or areas of interest that would be helpful for you to care for your child with constipation?

*Further probes if appropriate.*

- Give an explanation of what a “topic” is, if not understood. “For example a topic could be “what causes constipation” or “which medication is used”
- Do you think that there are any gaps in the information that you receive?

*Section 3: Usefulness of current information*

1. Which parts from the information, or topics, that you already receive do you find particularly useful?
2. Which parts are not useful?

*Further probes if appropriate.*

*Section 4: What format and content of information would caregivers like in the future?*

1. You may already have been given different information sources, what format has the information been in (e.g. leaflets, videos etc)?
2. Was the information that you have already received in a useful format?

- Probe – clarify what format is if not understood

1. Do you have a preference of format in which the information is provided?
2. What format of information would you find most helpful?
3. What formats of information are not helpful?
4. How easy has it been to access information about caring for your child?

*Further probes if appropriate*

*Summarising*

- In what way could the medical community best provide you with the information necessary to help you to support your child?
- Is there anything else you would like to add?
