# Supplementary material for: A “Curriculum of Information Needs” of Parents of Children With Chronic Constipation
Source: Clin Pediatr (Phila). 2025 Dec 1;65(3):403–10. doi: 10.1177/00099228251395563 (PMC12864524; doi:10.1177/00099228251395563)
Supplement: sj-docx-6-cpj-10.1177_00099228251395563 – Supplemental material for A “Curriculum of Information Needs” of Parents of Children With Chronic Constipation [file sj-docx-6-cpj-10.1177_00099228251395563.docx]

Round-1 - Interview Guide 2, for key stakeholders in Group 2

**Study title:** The information needs of caregivers of children with chronic constipation: a Delphi study

**Interview Documentation** (to be filled in by the interviewer during the interview)

*Interview information:*

Interview number:

Participant code number:

Date of interview:

Duration of interview:

Interviewer name:

*Demographics:*

Key stakeholder role:

Level of contact with caregivers with children with constipation, or soiling:

- Direct contact (speaking with them)
- Indirect contact

Further clarification:

Regularity of contact with children with constipation/soiling:

- Daily or almost daily
- A few times a week
- Once a week
- Once a month
- Very infrequently

**Guide**

*Introduction:*

1. Interviewer to introduce self, and ask participant what they would like to be called for the purposes of the interview, reminding them that their name will be removed from the transcript.
2. Interviewer to remind participant of the study title: *The information needs of caregivers of children with chronic constipation: a Delphi study.*
3. Interviewer to check that the participant has read and understood the information sheet,.
4. Interviewer to give participant an opportunity to ask any questions about their participation in the study.
5. Interviewer to remind participant that they are not obliged to answer any questions and are free to stop the interview at any point.
6. Interviewer to explain that the recording of the interview will begin, unless the participant requests otherwise, in which case detailed notes will be taken.
7. Interviewer to press record.
8. Consent process

*Section 1: Demographics:*

1. What is their role as a key stakeholder? (e.g. clinician, surgeon, role within ERIC)
2. What is their level of contact with caregivers with children with constipation, soiling or encoporesis? Direct or indirect? (Allow participant to expand on their answer)
3. What is the regularity of their contact with these caregivers?

*Section 2: Content of information which is currently provided*

We want to ensure that there is information on all the aspects of constipation that parents want to know about.

1. What do you think are the key topics or areas of interest that would be helpful for caregivers to support their child with constipation?

*Further probes if appropriate.*

- If not understood, give an explanation of what a “topic” is. For example a topic could be “what causes constipation” or “which medication is used”.
- Do you think that there are any gaps in the information that you have available to give to caregivers?

*Section 3: Usefulness of current information*

1. Thinking about the resources which are already available to the families that you look after, which of those are particularly useful?
2. Which parts are not useful?

*Further probes if appropriate.*

*Section 4: What format and content of information would caregivers like in the future?*

1. You may already have access to different information sources, what format has the information been in (e.g. leaflets, videos etc)?
2. Was the information that you have access to in a useful format?

- Probe – clarify what format is if not understood

1. Do families that you support seem to have a preference of format in which the information is provided?
2. What format of information would you find most helpful to share with families?=
3. What formats of information are not helpful to share with families?
4. How easy has it been to access these resources or share information with families?

*Further probes if appropriate*

*Summarising*

- In what way could the medical community best provide caregivers with the information necessary to help them support their child?
- Is there anything else you would like to add?
