# Supplementary material for: A “Curriculum of Information Needs” of Parents of Children With Chronic Constipation
Source: Clin Pediatr (Phila). 2025 Dec 1;65(3):403–10. doi: 10.1177/00099228251395563 (PMC12864524; doi:10.1177/00099228251395563)
Supplement: sj-docx-7-cpj-10.1177_00099228251395563 – Supplemental material for A “Curriculum of Information Needs” of Parents of Children With Chronic Constipation [file sj-docx-7-cpj-10.1177_00099228251395563.docx]

**Table: Supplementary data - Additional themes and codes**

| **Tag** | **Theme 2. Sources of current information** | **Frequency of code** |
| --- | --- | --- |
|  | **Sub-theme:** **2.1 Healthcare professionals** |  |
| 2.1.1 | Healthcare professionals in general | 104 |
| 2.1.2 | General Practitioner | 52 |
| 2.1.3 | Continence nurse | 5 |
| 2.1.4 | Pharmacist | 4 |
|  | **Sub-theme:** **2.2 Other Professionals** |  |
| 2.2.1 | Schools | 15 |
|  | **Sub-theme:** **2.3 Charity sector** |  |
| 2.3.1 | ERIC | 68 |
| 2.3.2 | Bowel and Bladder UK | 10 |
|  | **Sub-theme:** **2.4 Forums** |  |
| 2.4.1 | Other parents via forums (e.g. on Facebook) | 59 |
| 2.4.2 | Support groups | 17 |
|  | **Sub-theme:** **2.5 General** |  |
| 2.5.1 | Other online resources | 19 |
| 2.5.2 | Signposting from professionals | 16 |
|  | **Theme 3. Format / Media of information** |  |
|  | **Sub-theme:** **3.1 Verbal information** |  |
| 3.1.1 | Information provided verbally | 46 |
| 3.1.2 | Helpline / telephone support | 9 |
|  | **Sub-theme:** **3.2 Paper/printed** |  |
| 3.2.1 | Written information, leaflets | 116 |
| 3.2.2 | Children's books | 18 |
| 3.2.3 | Diagrams | 14 |
| 3.2.4 | Parent books | 5 |
|  | **Sub-theme:** **3.3 Digital** |  |
| 3.3.1 | Videos | 70 |
| 3.3.2 | Interactive resources | 51 |
| 3.3.3 | Single point of access for information | 36 |
| 3.3.4 | Apps (applications) | 36 |
| 3.3.4 | Online resources | 32 |
| 3.3.5 | QR codes or links (e.g. SMS) to websites | 27 |
| 3.3.6 | Social Media | 23 |
| 3.3.7 | Single summary | 5 |
| 3.3.8 | Email | 3 |
| 3.3.9 | TV | 1 |
|  | **Theme 4. Barriers to education** |  |
|  | **Sub-theme:** **4.1 Parental barrier** |  |
| 4.1.1 | Education level of parent | 107 |
| 4.1.2 | Motivation for education | 83 |
| 4.1.3 | Social factors | 56 |
| 4.1.4 | "Taboo" topic | 20 |
| 4.1.5 | Language | 12 |
| 4.1.6 | Time taken to review resources | 6 |
| 4.1.7 | Parent child relationship | 5 |
| 4.1.8 | Not pitched for appropriate ages | 3 |
|  | **Sub-theme:** **4.2 Healthcare barrier** |  |
| 4.2.1 | Late diagnosis | 23 |
| 4.2.2 | Funding and financial constraints | 22 |
| 4.2.3 | Waiting times to see professional | 19 |
| 4.2.4 | Incorrect information provided | 8 |
| 4.2.5 | Parents not being listened to/believed by HCPs | 5 |
| 4.2.6 | Lack of training for HCPs | 1 |
