# Supplementary material for: A “Curriculum of Information Needs” of Parents of Children With Chronic Constipation
Source: Clin Pediatr (Phila). 2025 Dec 1;65(3):403–10. doi: 10.1177/00099228251395563 (PMC12864524; doi:10.1177/00099228251395563)
Supplement: sj-pdf-3-cpj-10.1177_00099228251395563 – Supplemental material for A “Curriculum of Information Needs” of Parents of Children With Chronic Constipation [file sj-pdf-3-cpj-10.1177_00099228251395563.pdf]

UNIVERSITY OF LEEDS

Faculty of Medicine and Health, School of Medicine,
Leeds Institute for Health Sciences

Participant Information Sheet

Thank you for showing interest in our research project.

Study title: The information needs of caregivers of children with chronic constipation: a Delphi study

Our names are ***[anonymised for double peer review]*** and we are fourth year medical students from the University of Leeds. We are working collaboratively with the UK charity ERIC to undertake a piece of research to better help the parents, carers and children who have chronic constipation.

This information sheet will tell you more about the project and what you will be asked to do if you take part. Please read this sheet in full and take some time to consider whether you would like to be involved.

Background

The aim of this study is to explore the information needs of caregivers of children with chronic constipation.

Why have you been chosen?

You have been chosen because of your engagement with the charity ERIC, and due to your professional expertise in working with caregivers and children with childhood constipation.

This means that you can give your opinion on the information needs of caregivers of children with chronic constipation. Our findings will potentially help to increase the quality and quantity of information available for caregivers of children with constipation in the future.

Do you have to take part?

No, participation is voluntary.

If you would like to participate, we will ask you to confirm your consent at the start of the interview.

What will you be asked to do in the study?

You will be interviewed via Zoom for approximately 20 minutes, and definitely under 45 minutes.

The interview will involve a discussion on your opinions on the information needs for caregivers of children with constipation, what is already available and what you think is needed in the future including the methods of delivery for this content.

You are welcome to decline to answer any of the questions without incurring any negative consequences.

Who will be present during the interview?

The interview is a discussion between you and one of the researchers. No other interviewer or researcher will be present, but if you wished to have somebody else present for your own support then this is welcomed.

Will the interview be recorded?

Yes, this is to enable us to put the conversation into written words. The interview will be recorded on Zoom, an encrypted (secure) software, and only the researchers will have access to this recording before it is deleted post-transcription. Furthermore, identifiable information will be removed.

What are the benefits of taking part in the study?

Our results may help to identify common themes relating to the information needs for caregivers of children with constipation. This could help to identify what information needs to be produced and the future methods of delivery for this content. This could help caregivers of children with chronic constipation in the future to feel supported with high quality and appropriate information.

What will happen to the research results?

Any identifiable information will be anonymised.

After the data collection and anonymization process, data will be analysed and a summary report of key findings will be written. This report will be shared with the charity ERIC to enable ongoing work to better create resources for families.

Will your information be kept confidential?

Yes. The interviews will be digitally recorded and any identifiable information will be kept alongside your interview transcript and only the researcher will know the names of participants involved. Data will be stored on a secure password-protected OneDrive at the University of Leeds.

What should you do next if you want to be involved in the study?

If you have read and understood all of the information above, and you like to be involved in the research, please contact the researchers via the details below.

Contact information

If you would like to contact the researchers, with any questions at all, please send an email or give them a call on the number below:

Email address: ***[anonymised for double peer review]***

Supervisor details: ***[anonymised for double peer review]***

Thank you for taking the time to read this information sheet.
