# Supplementary material for: A “Curriculum of Information Needs” of Parents of Children With Chronic Constipation
Source: Clin Pediatr (Phila). 2025 Dec 1;65(3):403–10. doi: 10.1177/00099228251395563 (PMC12864524; doi:10.1177/00099228251395563)
Supplement: sj-pdf-4-cpj-10.1177_00099228251395563 – Supplemental material for A “Curriculum of Information Needs” of Parents of Children With Chronic Constipation [file sj-pdf-4-cpj-10.1177_00099228251395563.pdf]

Consent form (Round-1)

Faculty of Medicine and Health, School of Medicine, Leeds Institute for Health Sciences

UNIVERSITY OF LEEDS

Study title: The information needs of caregivers of children with chronic constipation: a Delphi study

Participant consent information

The text will be sent to the participants via email prior to the interview and the interviewer will then ask the questions in bold at the start of the interview.

Consent to take part in the following research project:

The information needs of caregivers of children with chronic constipation: A Delphi Study<br><br>Principal investigators: ***[anonymised for double peer review]***	Confirmation
**Have you read and understood the participant information sheet?**<br>I confirm that I have read and understand the participant information sheet explaining the above research project and I have had the opportunity to ask questions about the project.	
**Do you understand that your participation is voluntary and that you are free to withdraw at any time within 48 hours after the interview has finished?**<br>This is without giving any reason and without there being any negative consequences. I can withdraw by contacting ***[anonymised]*** up to 48 hours post-interview limit. Additionally, I understand that should I not wish to answer particular questions, I am free to decline.<br><br>Tabitha's contact details: ***[anonymised for double peer review]***<br>***[anonymised for double peer review]***<br>Sadia's contact details: ***[anonymised for double peer review]***<br>***[anonymised for double peer review]***<br><br>If I withdraw from the research within 48 hours post-interview, I understand that any of my personal data or interview responses will not be included in the research project.	
**Do you understand that your responses are kept confidential, will be stored and will be used by the research team?**<br>I give permission for members of the research team to have access to my anonymised responses. I understand that my name will not be linked with the research materials, and I will not be identified or identifiable in the report or reports that result from the research.<br>I understand that my responses will be kept strictly confidential.<br>I agree for the data collected from me to be stored and used in relevant future research in an anonymised form.	

I understand that other genuine researchers will have access to this data only if they agree to preserve the confidentiality of the information as requested in this form.<br>I understand that other researchers may use my words in publications, reports, web pages, and other research outputs, only if they agree to preserve the confidentiality of the information as requested in this form.	
**Do you understand that relevant sections of the data collected during the study, may be looked at by auditors from the University of Leeds?** If it is relevant to my taking part in this research. I give permission for these individuals to have access to my records.	
**Do you agree to take part in the above research project** and will inform the lead researcher should my contact details change during the project and, if necessary, afterwards.	
